# Parents, Children and the State

**Published:** 1909-02-27

**Authors:** 


### The Hospital

A Journal of

The Medical Sciences and Hospital Administration.

New Series. No. 104, Vol. IV. [No. 1176, Vol. XLV.] SATURDAY, FEBRUARY 27, 1909.

### PARENTS, CHILDREN AND THE STATE.

A contributor to a recent number of the *Westminster Review*, in an article devoted to the existing relations between children and the State, is at much pains to show that the present rate of infantile mortality is decreased by evolution. Now the " survival of the fittest " is a fine explanatory thesis which has explained much in the almost infinite past of the animal and vegetable creations, and will no doubt be continuing its explanations ages hence. But the truth to which it gives expression cannot be admitted as an acceptable guide of human action in relation to human society. Nature, exercising a breadth of view which we may admire but cannot imitate, treats the individual with undisguised contempt. To her he is almost nothing, but the welfare of the race is all. The individual is an item in the continuance of his race, and to that end he claims notice, but hardly beyond it. Sparta had the lesson from Nature, took it to heart, destroyed its weaklings, and built up a race which, small as it was, has left no inconsiderable mark on history. But that is an old story. The inveterate pessimist may sigh for those hard old day of giants, but as in all probability he will be sighing in an armchair, before a good fire, with his feet in carpet slippers, and with no immediate prospect of any greater hardship than cold sheets when he goes to bed, his distresses need not vex us. Ours is an age of Individualism in its widest sense. Perhaps we are wrong, and, given a wider horizon, should follow Nature's lead. But we, or most of us, cannot see very far, and thus the health of the parish bull to-day is of more moment than the status of the British Empire a couple of centuries hence. We are far from saying that this limitation of vision is ideal, but it exists, and dominates the situation.

It is no longer possible, therefore, to gloze the colossal figures of our infantile mortality with a thread-bare tag of evolutionary philosophy. What, then, remains to do? " Of every 1,000 infants born," says the writer in the *Westminster Review*, " 150 die before they reach the age of one year, and the majority of the 150 die from causes which in the present state of medical knowledge are either remediable or removable. Among these causes may be mentioned the evil effects of slum life, the industrial employment of child-bearing women, and insufficient and unsuitable food." True indeed! If every child-bearing woman now in slum-land had a cottage in the country and two hundred a year, there is no doubt at all that the undertakers would suffer. But not altogether for the reason which might at first sight appear. Two hundred a year brings with it a sense of responsibility which twenty shillings a week (if times are good) is quite unable to evoke; and by hook, or it may be by crook, two hundred a year contrives that hostages to fortune shall not be so numerous as we find them in the slums. There is neither wisdom nor policy in blinking the fact that families shrink as means increase. The reason is not far to seek. In the first place, a higher standard of living means later and less prolific marriages, more consideration for the future of offspring, and some sense of conscience touching the procreation of children for whom no provision can be made. It means, too, an appreciation of the fact that children have to be educated out of the private purse, as well as clothed and fed. But in slum-land no thought for the morrow comes to temper indulgence. Prudence is undreamt of, or limits itself to the provision of a small insurance to cover the funeral expenses of the latest arrival upon the squalid stage. To talk of parental responsibility in such a connection is a bitter jest; for the thing does not exist or the state of affairs could not. Yet this same parental responsibility is undeniably a saving grace. The writer in the *Westminster Review* admits it, as who would not! How is it to be compassed? Shall we compass it by daily diminishing the calls which, in nature, a child has a right to make upon his begetters? Shall we compass it by saying, " Be fruitful and multiply and care not, for the savings of the prudent are saved for you? " No, indeed!

We are faced, then, with this dilemma. Children of the slums must die in large numbers unless the State takes them into its protection. *Per contra*, if the State does this thing, parental responsibility (when it exists in the slums) is diminished to extinction, and no considerations of prudence remain as an obstacle to improvident marriages and the production of large families. In consequence the burden of the

State may be expected to grow by leaps and bounds, while the parents under such a *régime* are only parents in the barest animal sense. Between these two things we have been called upon to choose, and by virtue of our ingrained respect for the individual we have chosen, and continue to choose, the former. We do not say wrongly, for the practical needs of daily life commonly drive us to select an alternative which armchair philosophy repudiates. The children are there, and no cataract of pious wishes can unmake them. If they are not cared for they either die, in which case the instinct of humanity rebels; or they live to grow up weaklings or wastrels, and in either event are not only a loss to the efficiency of the State, but an actual drag and charge upon its more efficient members. Our field of vision is limited, as has been said, and we cannot see far. It were wiser, under the circumstances, not to look even as far as we think we can, lest we be disheartened by the apparent prospect. Nevertheless apparent prospects are often deceptive, and it may not pass the wit of our law-givers to evolve some means of reconciling a human care for the living with the best interests of the stock. Certainly, so far as we can see, they have a problem before them.

